# Complete plastome sequences of *Equisetum arvense *and *Isoetes flaccida*: implications for phylogeny and plastid genome evolution of early land plant lineages

**DOI:** 10.1186/1471-2148-10-321

**Published:** 2010-10-23

**Authors:** Kenneth G Karol, Kathiravetpillai Arumuganathan, Jeffrey L Boore, Aaron M Duffy, Karin DE Everett, John D Hall, S Kellon Hansen, Jennifer V Kuehl, Dina F Mandoli, Brent D Mishler, Richard G Olmstead, Karen S Renzaglia, Paul G Wolf

**Affiliations:** 1 The Lewis B. and Dorothy Cullman Program for Molecular Systematics Studies, The New York Botanical Garden, Bronx, New York 10458, USA; 2 Benaroya Research Institute at Virginia Mason, 1201 Ninth Avenue, Seattle, WA 98101, USA; 3 Department of Energy Joint Genome Institute, 2800 Mitchell Drive, Walnut Creek, CA 94598, USA; 4 Genome Project Solutions, 1024 Promenade Street, Hercules, CA 94547, USA; 5 Ecology Center and Department of Biology, Utah State University, Logan, UT 84322, USA; 6 Department of Biology, University of Washington, Seattle, WA 98195, USA; 7 Physical Biosciences, Lawrence Berkeley National Laboratory, 1 Cyclotron Rd, Berkeley, CA. 94720 USA; 8 4500 NE 40th Street, Seattle, WA 98105, USA; 9 Department of Integrative Biology and University and Jepson Herbaria, 1001 Valley Life Sciences Bldg., University of California, Berkeley, CA 94720, USA; 10 Southern Illinois University, Plant Biology-SIUC, Mailcode: 6509, Carbondale, IL 62901, USA

## Abstract

**Background:**

Despite considerable progress in our understanding of land plant phylogeny, several nodes in the green tree of life remain poorly resolved. Furthermore, the bulk of currently available data come from only a subset of major land plant clades. Here we examine early land plant evolution using complete plastome sequences including two previously unexamined and phylogenetically critical lineages. To better understand the evolution of land plants and their plastomes, we examined aligned nucleotide sequences, indels, gene and nucleotide composition, inversions, and gene order at the boundaries of the inverted repeats.

**Results:**

We present the plastome sequences of *Equisetum arvense*, a horsetail, and of *Isoetes flaccida*, a heterosporous lycophyte. Phylogenetic analysis of aligned nucleotides from 49 plastome genes from 43 taxa supported monophyly for the following clades: embryophytes (land plants), lycophytes, monilophytes (leptosporangiate ferns + *Angiopteris evecta *+ *Psilotum nudum *+ *Equisetum arvense*), and seed plants. Resolution among the four monilophyte lineages remained moderate, although nucleotide analyses suggested that *P. nudum *and *E. arvense *form a clade sister to *A. evecta *+ leptosporangiate ferns. Results from phylogenetic analyses of nucleotides were consistent with the distribution of plastome gene rearrangements and with analysis of sequence gaps resulting from insertions and deletions (indels). We found one new indel and an inversion of a block of genes that unites the monilophytes.

**Conclusions:**

Monophyly of monilophytes has been disputed on the basis of morphological and fossil evidence. In the context of a broad sampling of land plant data we find several new pieces of evidence for monilophyte monophyly. Results from this study demonstrate resolution among the four monilophytes lineages, albeit with moderate support; we posit a clade consisting of Equisetaceae and Psilotaceae that is sister to the "true ferns," including Marattiaceae.

## Background

Patterns and processes of organic evolution are reflected in the structure and sequences of organisms' genomes. Although we are only starting to accumulate sufficient data to compare nuclear genomes of plants, more data are available for the smaller plastid genomes (plastomes). Comparative work on plastomes began in the early 1980's using restriction site mapping and hybridization with heterologous probes to generate phylogenetically informative data within small clades, generally below the family level [1,2] as well as using comparative mapping to examine differences among more distantly related groups [3-6]. By the 1990's the emphasis shifted to nucleotide sequences from targeted regions (genes) with the result that studies with broad genomic sampling were few. However, a reduction in sequencing cost and concomitant development of analytical tools over the past 10 years has resulted in resurgence of comparative plastid genomics.

The plastome is highly conserved in overall structure and this provides the basis for comparative studies [4]. However, there is still sufficient variation to identify rare genomic events that mark critical branches in land plant evolution, thereby elucidating early evolutionary modifications to the plastid genome [e.g., [5,7]]. Plastomes of *Marchantia polymorpha *[8] and *Nicotiana tabacum *[9] were the first to be sequenced and within 19 years there were 45 complete plastome sequences for green plants (Viridiplantae) in GenBank [10]. Acceleration of efforts yielded 146 plastome sequences by the end of 2009, with many others in progress.

Complete genome sequences offer several advantages over restriction site maps and nucleotide sequences of targeted regions. In addition to substantially increasing the number of gene sequences for comparison, plastome sequences provide vast structural and evolutionary information that includes gene order, genome rearrangements, patterns of base pair composition, codon usage, mechanisms of gene duplication and gene loss (e.g., pseudogenization), patterns of nucleotide insertion and deletion (indels), and the occurrence of noncoding regions (such as plastome microsatellites and introns). We now have sufficient sampling from most major land plant clades to begin comparative analyses of their plastomes in earnest.

Despite considerable progress over the last 15 years in our understanding of green plant phylogeny, several nodes remain poorly resolved. One example is the monilophytes, which include five major lineages: leptosporangiate ferns, horsetails, marattioid ferns, ophioglossoid ferns and psilophytes. The monilophytes seem to be well-supported as a group, and are generally accepted as sister to seed plants [11-14]. But relationships among the major monilophyte lineages remain unclear. Here we present the complete plastome for the horsetail *Equisetum arvense *L., representing one of the last major monilophyte lineages to have a representative complete plastome available. For further resolution of land plant relationships and plastome evolution we also sequenced the plastome of *Isoetes flaccida *Shuttlw. ex A. Braun, the last of the three major lycophyte lineages to be sampled. We use data for 49 genes from 43 green plant taxa to infer phylogenetic relationships of land plants and compile information on the distribution of gene translocations, genomic inversions, gene content and indels to augment the phylogenetic signal from gene variation. We also examine codon usage and base composition. Patterns of plastome architecture are compared across early land plant lineages that diverged 400 to 500 million years ago.

## Results and Discussion

Our analyses included representatives of all major land plant lineages as well as sampling of charophycean green algae for appropriate phylogenetic context. We do not provide detailed comparative plastome analyses for charophycean algae and seed plants because these are presented elsewhere: charophycean green algae [15,16], gymnosperms [17-20] and angiosperms [21-23].

### Plastome structures and composition

**Figure 1:**
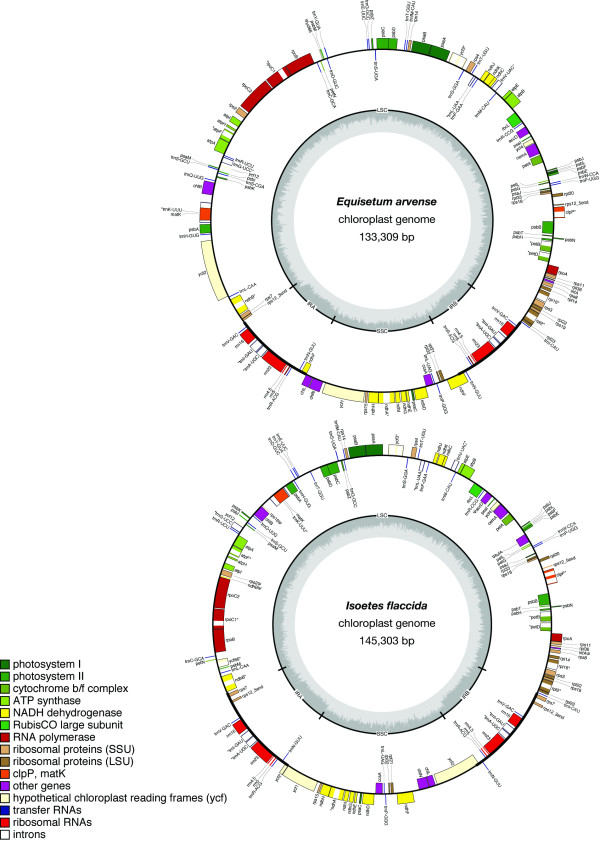
**Gene maps of the *Equisetum arvense *and *Isoetes flaccida *plastomes**. The inverted repeats (IRA and IRB) which separate the genome into the large (LSC) and small (SSC) single copy regions are indicated on the inner cycle along with the nucleotide content (G/C dark grey, A/T light grey). Genes shown on the outside of the outer circle are transcribed clockwise and those on the inside counter clockwise. Gene boxes are color coded by functional group as shown in the key. An asterisk (*) denotes genes with introns and a psi (ψ) denotes pseudogenes.

Gene maps for plastomes of *Equisetum arvense *and *Isoetes flaccida *are shown in Fig. 1. The complete plastome of *E. arvense *is 133,309 base pairs (bp) and includes a 93,542 bp large single-copy (LSC) region, a 19,469 bp small single-copy (SSC) region and two 10,149 bp inverted repeats (IRA and IRB). The complete plastome of *I. flaccida *is 145,303 bp and includes a 91,862 bp LSC, a 27,205 bp SSC and two 13,118 bp IRs. The overall G/C content is 33.36% for *E. arvense *and 37.94% for *I. flaccida*. Both annotated plastomes have been deposited in GenBank (Table 1).

**Table 1:** List of plastomes analyzed in this study.

Taxon	GenBank Accession	Plastome size (bp), G/C (%), ENc	Citation
**Angiosperms**:			
*Arabidopsis thaliana*	NC_000932	154478, 40.3, 47.90	Sato *et al.* 1999 [68]
*Morus indica*	NC_008359	158484, 40.5, 47.96	Ravi *et al.* 2007 [69]
*Vitis vinifera*	NC_007957	160928, 41.0, 48.64	Jansen *et al.* 2006 [70]
*Nicotiana tabacum*	NC_001879	155943, 41.0, 48.79	Shinozaki *et al.* 1986 [9]
*Helianthus annuus*	NC_007977	151104, 40.8, 48.56	Timme *et al.* 2007 [71]
*Spinacia oleracea*	NC_002202	150725, 40.7, 48.25	Schmitz-Linneweber *et al.* 2001 [72]
*Ranunculus macranthus*	NC_008796	155129, 41.4, 49.68	Raubeson *et al.* 2007 [23]
*Agrostis stolonifera*	NC_008591	136584, 41.2, 49.76	Saski *et al.* 2007 [73]
*Lemna minor*	NC_010109	165955, 40.1, 46.69	Mardanov *et al.* 2008 [74]
*Phalaenopsis aphrodite*	NC_007499	148964, 40.4, 47.83	Chang *et al.* 2006 [75]
*Dioscorea elephantipes*	NC_009601	152609, 40.5, 47.73	Hansen *et al.* 2007 [21]
*Acorus calamus*	NC_007407	153821, 41.7, 49.76	Goremykin *et al.* 2005 [43]
*Drimys granadensis*	NC_008456	160604, 41.6, 49.82	Cai *et al.* 2006 [76]
*Liriodendron tulipifera*	NC_008326	159886, 41.8, 49.67	Cai *et al.* 2006 [76]
*Illicium oligandrum*	NC_009600	148553, 42.1, 50.37	Hansen *et al.* 2007 [21]
*Chloranthus spicatus*	NC_009598	157772, 41.6, 49.65	Hansen *et al.* 2007 [21]
*Nymphaea alba*	NC_006050	159930, 42.2, 50.68	Goremykin *et al.* 2004 [77]
*Amborella trichopoda*	NC_005086	162686, 41.7, 49.66	Goremykin *et al.* 2003 [78]
**Gymnosperms**:			
*Pinus thunbergii*	NC_001631	119707, 41.4, 48.98	Wakasugi *et al.* 1994 [79]
*Ephedra equisetina*	NC_011954	109518, 37.5, 44.35	Wu *et al.* 2009 [19]
*Gnetum parvifolium*	NC_011942	114914, 40.2, 53.80	Wu *et al.* 2009 [19]
*Welwitschia mirabilis*	NC_010654	119726, 38.5, 45.79	Wu *et al.* 2009 [19]
*Cycas taitungensis*	NC_009618	163403, 41.7, 48.58	Wu *et al.* 2007 [20]
*Ginkgo biloba*	various	N. A., 41.8, 48.87	Leebens-Mack *et al.* 2005 [80]
**Monilophytes:**			
*Adiantum capillus-veneris*	NC_004766	150568, 44.2, 55.12	Wolf *et al.* 2003 [37]
*Alsophila spinulosa*	NC_012818	156661, 43.6, 52.99	Gao *et al.* 2009 [38]
*Angiopteris evecta*	NC_008829	153901, 39.2, 44.32	Roper *et al.* 2007 [27]
*Psilotum nudum*	NC_003386	138829, 39.3, 45.80	GenBank direct submission
*Equisetum arvense*	GU191334	133309, 37.2, 41.94	This study
**Lycophytes:**			
*Selaginella uncinata*	AB197035	144170, 54.3, 58.30	Tsuji *et al.* 2007 [35]
*Selaginella moellendorffii*	FJ755183	143780, 51.2, 57.73	GenBank direct submission
*Isoetes flaccida*	GU191333	145303, 41.9, 48.84	This study
*Huperzia lucidula*	NC_006861	154373, 40.3, 46.66	Wolf *et al.* 2005 [39]
**Bryophytes:**			
*Anthoceros formosae*	NC_004543	161162, 38.2, 42.21	Kugita *et al.* 2003 [30]
*Syntrichia ruralis*	FJ546412	122630, 34.5, 35.46	Oliver *et al.* (in press) [32]
*Physcomitrella patens*	NC_005087	122890, 35.1, 36.12	Sugiura *et al.* 2003 [33]
*Marchantia polymorpha*	NC_001319	121024, 34.0, 35.25	Ohyama *et al.* 1986 [8]
**Charophyte Algae:**			
*Staurastrum punctulatum*	NC_008116	157089, 37.1, 38.28	Turmel *et al.* 2005 [81]
*Zygnema circumcarinatum*	NC_008117	165372, 39.2, 42.45	Turmel *et al.* 2005 [81]
*Chaetosphaeridium globosum*	NC_004115	131183, 35.0, 36.95	Turmel *et al.* 2002 [82]
*Chara vulgaris*	NC_008097	184933, 35.7, 39.57	Turmel *et al.* 2006 [83]
*Chlorokybus atmophyticus*	NC_008822	152254, 39.1, 43.85	Lemieux *et al.* 2007 [15]
*Mesostigma viride*	NC_002186	118360, 34.9, 34.83	Lemieux *et al.* 2000 [84]

GenBank accession numbers, plastome sizes in base pairs (bp), percent G/C content, effective number of codons (ENc) and original citations are shown.

### Gene content of *Isoetes *and *Equisetum*

Several minor differences in gene content between *Equisetum arvense *and *Isoetes flaccida *were identified (Table 2). We detected *trnS-CGA *only in *E. arvense, *and pseudogenes of *rps16, tufA *and an extra fragment of *ndhB *only in *I. flaccida*. Because of difference in the IR boundaries *E. arvense *has one copy of *rps7 *whereas *I. flaccida *has two copies. The position of the IR boundary also results in a small fragment of *ycf2 *in the IR of *I. flaccida*. The gene *ycf66 *is in both taxa, but appears to be pseudogenized in *E. arvense*. The genes *accD, infA *and *rps2 *are also in both taxa but appear to be pseudogenized in *I. flaccida*.

**Table 2:** List of plastid genes and pseudogenes annotated for *Equisetum arvense *and *Isoetes flaccida*.

Gene class				
**Ribosomal RNAs**	*rrn4.5 *x2	*rrn5 *x2	*rrn16 *x2	*rrn23 *x2
**Transfer RNAs**	trnA-UGC* x2	*trnC-GCA*	*trnD-GUC*	*trnE-UUC*
	*trnF-GAA*	*trnG-GCC*	trnG-UCC*	*trnH-GUG*
	*trnI-CAU*	trnI-GAU* x2	trnK-UUU*	*trnL-CAA*
	trnL-UAA*	*trnL-UAG*	*trnM-CAU*	*trnfM-CAU*
	*trnN-GUU *x2	*trnP-GGG*	*trnP-UGG*	*trnQ-UUG*
	*trnR-ACG *x2	*trnR-CCG *c	*trnR-UCU*	*trnS-CGA *in *Ea*
	*trnS-GCU*	*trnS-GGA*	*trnS-UGA*	*trnT-GGU*
	*trnT-UGU*	*trnV-GAC *x2	trnV-UAC*	*trnW-CCA*
	*trnY-GUA*			
**Photosystem I**	***psaA***	***psaB ***b	***psaC***	***psaI ***b
	***psaJ***	*psaM*		
**Photosystem II**	***psbA***	***psbB***	***psbC***	***psbD***
	***psbE***	***psbF***	***psbH ***b	***psbI***
	***psbJ***	***psbK***	***psbL ***b	***psbM***
	***psbN***	***psbT ***b	***psbZ***	
**Cytochrome**	***petA***	petB* b, e	petD* b, e	***petG ***b
	***petL***	*petN *b		
**ATP synthase**	***atpA***	***atpB***	***atpE***	atpF*
	***atpH***	***atpI ***b		
**Rubisco**	***rbcL ***b			
**Chlorophyll biosynthesis**	*chlB*	*chlL *b	*chlN*	
**NADH dehydrogenase**	ndhA* b	ndhB*	*ndhC*	*ndhD *b, i
	*ndhE*	*ndhF *i	*ndhG*	*ndhH *b
	*ndhI*	*ndhJ *i, e	*ndhK*	
**Ribosomal proteins**	rpl2*	***rpl14 ***b, i, e	rpl16*	***rpl20 ***b
	*rpl21 *i	*rpl22*	*rpl23*	*rpl32*
	*rpl33*	***rpl36***	*rps2 *ψ (If)	***rps3***
	***rps4 ***b	***rps7 ***x2 in *If*	***rps8 ***b	***rps11 ***b, s
	*rps12 *x2 tr	***rps14***	*rps15*	*rps16 *ψ in *If*
	***rps18***	***rps19***		
**RNA polymerase**	*rpoA *i	***rpoB ***i	rpoC1* i	*rpoC2 *i
**Miscellaneous proteins**	*infA *ψ (*If*)	*ccsA *b	*matK*	clpP*
	*accD *ψ (*If*), ss	*cemA *i	*tufA *ψ in *If*	
**Hypothetical proteins**	*ycf1 *b	*ycf2 *i	ycf3*	***ycf4 ***b
	*ycf12*	ycf66* ψ (*Ea*)		
**Duplicate gene fragments**	*ndhB *ψ in *If*	*ycf2 *IR in *If*	*ndhF *IR in *Ea*	

* = intron-containing gene; tr = trans-spliced. In *If *= gene in *I. flaccida *but not *E. arvense*; in *Ea *= gene in *E. arvense *but not *I. flaccida*. Hypothesized RNA editing in *I. flaccida*: b = start edited; i = internal stop codon; e = stop codon edited; c = anticodon of tRNA edited. In *E. arvense*: s = start edited; ss = start and stop edited. ψ (If) = pseudogene in *I. flaccida *and gene present in *E. arvense *but not as pseudogene. ψ in If = pseudogene in *I. flaccida *but gene absent in *E. arvense*. ψ (Ea) = pseudogene in *E. arvense *and gene present in *I. flaccida *but not as pseudogene. Note that *rps7 *is in the IR in *I. flaccida *so has two copies (x2) but only one copy in *E. arvense*. Genes in bold were those used for phylogenetic analyses.

#### clpP introns

In our newly described plastomes, *clpP *contains only one intron in *Equisetum arvense *and two introns in *Isoetes flaccida*. The distribution of introns in *clpP *is variable across plastomes sampled to date: all bryophytes have two introns, lycophytes have either two introns (*Huperzia lucidula *and *I. flaccida*) or one intron (*Selaginella moellendorffii *and *S. uncinata*), and all monilophytes except *E. arvense *have two introns. Among gymnosperms, *Cycas taitungensis *has two introns and other gymnosperms have either lost both introns (*Pinus thunbergii*, *Ephedra equisetina *and *Welwitschia mirabilis*) or have lost *clpP *entirely (*Gnetum parvifolium*). Most flowering plant *clpP *genes have two introns, except the monocot *Agrostis calamus*. In the charophycean algae, *Chara vulgaris *and *Chaetosphaeridium globosum *each have one *clpP *intron, whereas other charophycean green algae have no introns in *clpP*. It appears that the second *clpP *intron is unique to land plants, although there have been several lineage-specific losses (e.g., *Selaginella *spp., *E. arvense*, most gymnosperms, and at least one lineage of monocots).

#### tufA in land plant plastomes

The functional *tufA *gene is encoded in the plastid genome of most green algae but has been transferred to the nuclear genome in land plants and certain charophycean green algae [24-26]. A *tufA*-like fragment was identified in *Anthoceros formosae*, cycads and *Gingko biloba *plastomes situated between *psbE *and *petL *[20]. We found a 1,364-bp region between *psbE *and *petL *in *Isoetes flaccida *that shares 41% nucleotide similarity with the plastid encoded *tufA *gene found in *Chara vulgaris*. The *I. flaccida tufA *homologue lacks the expected start and stop codons and contains 29 internal stop codons within the reading frame. Furthermore, when this region was aligned with the *C. vulgaris *homologue, 38 indels were identified (Additional File 1). Taken together, the *I. flaccida tufA*-like region is most likely a pseudogene. These relictual fragments of a once functional plastid gene now identified in four distantly related land plant lineages (i.e., hornworts, lycophytes and two gymnosperms) offer an interesting opportunity to study patterns of plastome gene loss as well as the process of gene decay.

#### Putative RNA editing

Reliable inferences on RNA editing require comparisons of genomic sequence to that of mature mRNA or cDNA. However, a fraction of editing sites are in start or stop codons, or result in premature stop codons, thus providing a hint of RNA editing from genomic sequences. We found such evidence in two genes in *Equisetum arvense *and 28 genes in *Isoetes flaccida *(Table 2). The former had an undetermined start codon for *rps11 *and undetermined start and stop codons for *accD*. In *I. flaccida *we found 21 loci with undetermined start codons, two with undetermined stop codons, and seven internal stop codons. Based on the sequence of *trnR-CCG*, we also infer that the second position of the anticodon is RNA edited (U- >C).

#### ycf1

The region between *chlN *and *rps15 *in plastomes presents an interesting challenge. In certain taxa this region contains two putative protein-coding genes, both read from the same DNA strand, but in different reading frames. Other taxa, including *Equisetum arvense *and *Isoetes flaccida, *contain a single large open reading frame (orf) commonly annotated as *ycf1*. Recent interpretation of the interruption of *ycf1 *has been either to recognize the entire region as a *ycf1 *pseudogene [e.g., [27]], or to recognize the larger of the two orfs as *ycf1 *while leaving the smaller unannotated [e.g., [28]]. The function of these orfs (or orf) is unknown and there are at least two processes that explain the differences in putative orfs in this region of the plastome. One possibility is that the large orf could yield two separate protein products through a post-transcription edit that adds a stop codon. Another possibility is that the two-orf pattern is a result of a frame shift that can be read through during translation, yielding a single protein product [e.g., [29]]. However, transcripts of this region have been determined for *Anthoceros formosae *with two-orfs and *Adiantum capillus-veneris *with a single large orf [30,31]. These transcript data were consistent with the original DNA annotation. That is, the reading frame was not 'corrected' in *Anthoceros formosae *and an internal stop codon was not detected in the larger orf in *Adiantum capillus-veneris*. Therefore at both the DNA and the RNA transcript levels there appears to be two alternative patterns.

### Gene order

**Figure 2:**
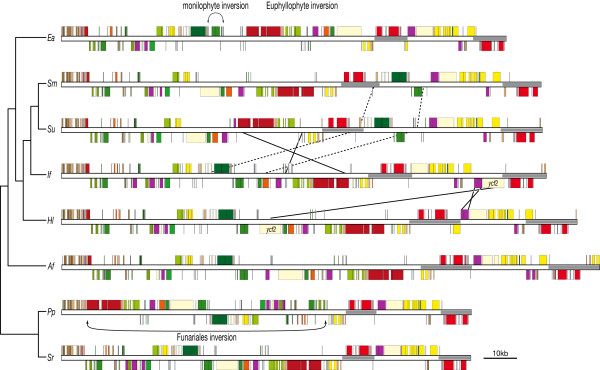
**Comparison of plastome maps of early diverging land plant lineages showing major structural changes**. Two mosses (*Pp *= *Physcomitrella patens *and *Sr *= *Syntrichia ruralis*), a hornwort (*Af *= *Anthoceros formosae*), four lycophytes (*Hl *= *Huperzia lucidula, If = Isoetes flaccida, Sm = Selaginella moellendorffii *and *Su = Selaginella uncinata*) and a monilophyte (*Ea *= *Equisetum arvense*) are compared. The liverwort *Marchantia polymorpha *shares a similar gene order with *Sr*, *Af *and *Hl *(not shown). Inverted repeat regions are depicted with grey boxes. Inversions and translocations are shown with arrows and solid or dashed lines respectively. The monilophyte-specific inversion shown for *Ea *is detailed in Fig. 3. Gene colors follow the key in Fig. 1.

Comparative plastome maps for representative early diverging land plant lineages are shown in Fig. 2. The liverwort *Marchantia polymorpha *was not included in this figure because it shares similar gene order with the moss *Syntrichia ruralis *and the hornwort *Anthoceros formosae*.

#### Bryophytes

Previous reports of plastomes representing exemplar bryophytes (liverwort *Marchantia polymorpha*, mosses *Physcomitrella patens *and *Syntrichia ruralis*, hornwort *Anthoceros formosae*) have shown that plastome content and gene order are highly conserved [8,30,32,33]. This is true even in *Aneura mirabilis*, the only non-photosynthetic bryophyte [28]. One exception was reported in *P. patens*, involving a large inversion (~71 kb) encompassing nearly the entire LSC relative to a sample of four other mosses [33]. Goffinet *et al.*[34] surveyed the phylogenetic extent of the *P. patens *inversion and found it was restricted to the moss order Funariales. Oliver *et al.*[32] found that the moss *S. ruralis *does not have this large inversion but instead has the ancestral land plant plastome gene order.

#### Lycophytes

The plastomes of *Isoetes flaccida *and *Huperzia lucidula *(lycophytes) share similar gene order with bryophytes. However, we identified two unique structural changes in *I. flaccida *(Fig. 2). First, *ycf2 *in *I. flaccida*, which normally resides in the LSC in most land plant plastomes, has been translocated to the SSC with the 5' end now incorporated into the IR. Second, the *chlL*/*chlN *gene cluster has been inverted in *I. flaccida *and now resides adjacent to *ycf2 *rather than *ycf1 *as in *H. lucidula*. Neither the *ycf2 *translocation nor the *chlL*/*chlN *inversion occurs in either of the *Selaginella *plastomes.

Both *Selaginella *plastomes are considerably different in gene order from typical land plant plastomes (Fig. 2) [[35], Banks et al. personal communications]. A large region (~14-kb) has been translocated from the LSC to the IR/SSC in both plastomes, although the genes included in this translocation differ slightly. In both *Selaginella *plastomes, *rps4 *marks one endpoint of this translocated segment and this gene now resides in the IR. The other endpoint resides in the SSC and is marked by *psbD *in *S. moellendorffii *and by three additional genes (*trnE-UUC*, *trnY-GUA *and *trnD-GUC*) in *S. uncinata*. These tRNA genes remain in the LSC adjacent to *ycf2 *in *S. moellendorffii*, as is the case in most land plant plastomes. Several unique features were identified in the plastome of *S. uncinata *including a ~20-kb LSC inversion (*psbI *to *rpoB*-*trnC-GAC*), duplication of *psbK *and *trnQ-UUG*, and translocation of *petN *from the LSC to the SSC [35].

#### Euphyllophytes

Raubeson and Jansen [5] identified a ~30-kb LSC inversion marking the euphyllophytes as a monophyletic group to the exclusion of the lycophytes. This inversion is confirmed in all monilophyte (including *Equisetum arvense*, Fig. 2) and seed plant plastomes sequenced and does not occur thus far in bryophyte or lycophyte plastomes (including *Isoetes flaccida*, Fig. 2).

#### Monilophytes

**Figure 3:**
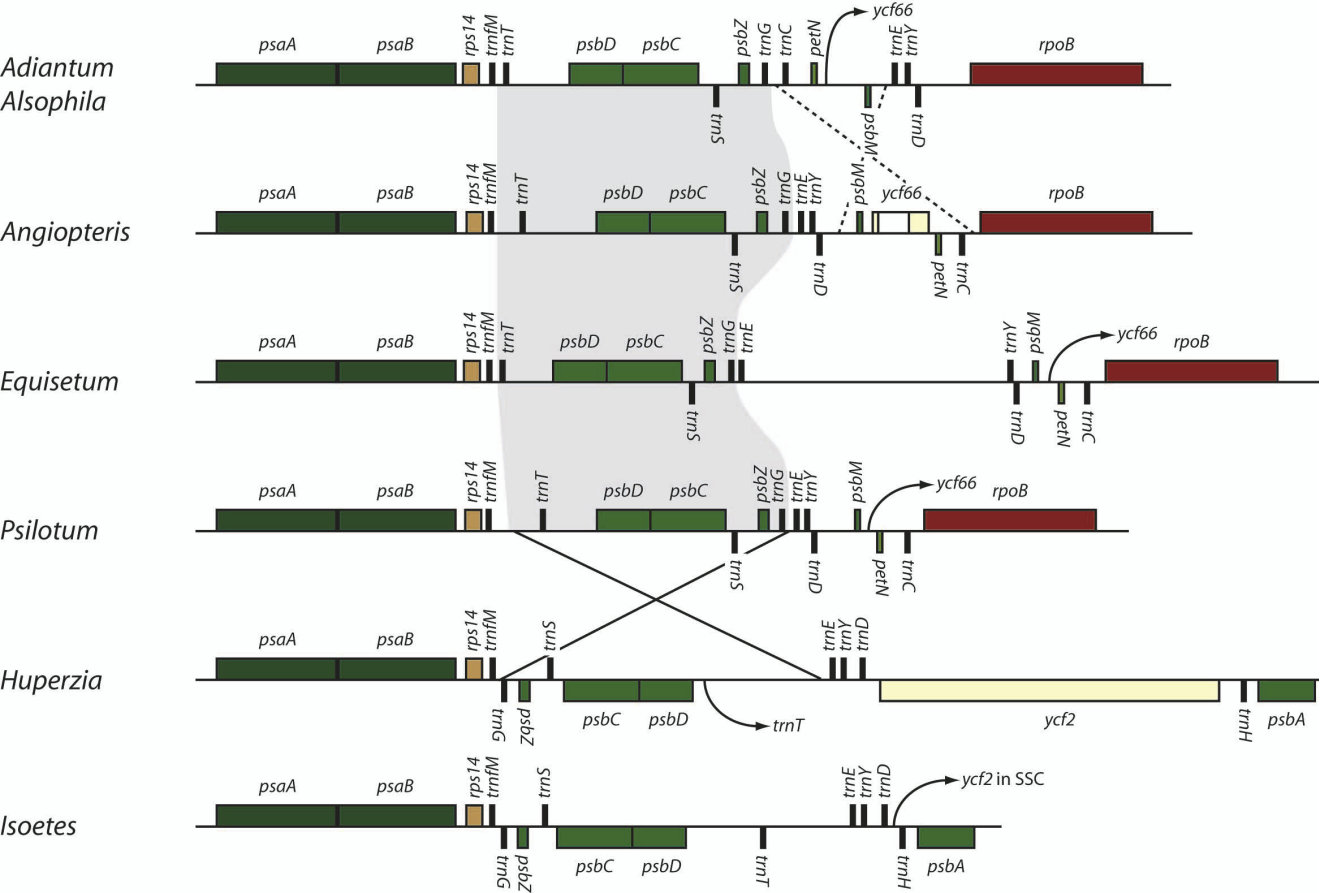
**Monilophyte-specific inversion**. Detailed view of an inversion found only in monilophyte taxa (solid black lines and grey shadow) and a second inversion found only in *Adiantum capillus-veneris *and *Alsophila spinulosa *(dashed black lines), which together represent a large clade comprising about 90% of extant fern species diversity. Additional gene loss and movements are shown with arrows (SSC = gene translocated to small single copy). The lycophyte gene order and orientation in this region is similar to that of bryophytes and seed plants (not shown). Note that the ~5 kb region between *trnE *and *trnY *unique to *Equisetum arvense *contains no significant open reading frames when subjected to ORF Finder (http://www.ncbi.nlm.nih.gov/gorf/gorf.html). Gene colors follow the key in Fig. 1.

Taking into account lineage specific gene losses and differences in IR gene content, *Angiopteris evecta*, *Equisetum arvense *and *Psilotum nudum *share identical gene order. Two large inversions associated with the IR and two smaller inversions in the LSC characterize the plastome of *Adiantum capillus-veneris *[36,37]. The tree fern *Alsophila spinulosa *shares these inversions [38], indicating that they probably occurred in the ancestor to a clade that includes most extant leptosporangiate ferns. Comparative gene-order analyses identified one of the LSC inversions (involving *trnG-GCC *to *trnT-GGU*) in *Psilotum nudum*, *Angiopteris evecta *and leptosporangiate ferns [27,38,39]. Here we identified the same inversion in *Equisetum arvense*, further supporting the hypothesis that this inversion is shared by all extant monilophyte taxa and can serve as a reliable molecular marker for the monophyly of this lineage (Fig. 3). Neither of the two IR inversions nor the remaining LSC inversion found in *Adiantum capillus-veneris *and *Alsophila spinulosa *was identified in other monilophyte plastomes.

#### The inverted repeat boundaries

**Figure 4:**
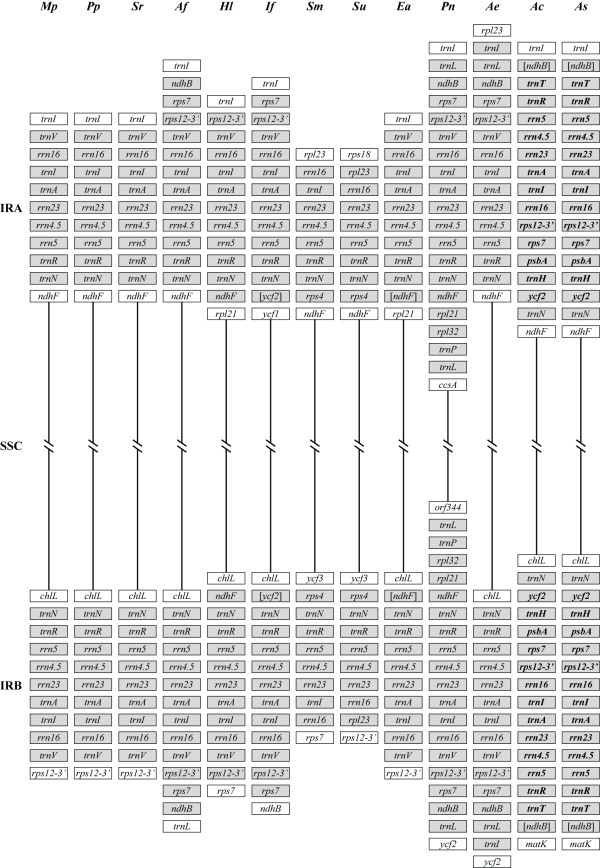
**Genes found in the inverted repeat of seed-free land plants**. A liverwort (*Mp *= *Marchantia polymorpha*), two mosses (*Pp *= *Physcomitrella patens *and *Sr *= *Syntrichia ruralis*), a hornwort (*Af *= *Anthoceros formosae*), four lycophytes (*Hl *= *Huperzia lucidula, If = Isoetes flaccida, Sm = Selaginella moellendorffii *and *Su = Selaginella uncinata*) and five monilophytes (*Ac *= *Adiantum capillus-veneris*, *Ae *= *Angiopteris evecta*, *As *= *Alsophila spinulosa*, *Ea *= *Equisetum arvense*, *Pn *= *Psilotum nudum*) are compared for inverted repeat (IR) gene content, including new plastomes reported here. The figure is organized so that IRA, the small single copy (SSC) and IRB are presented top to bottom. Grey boxes denote genes found in the IR and white boxes denote genes found in the large single copy or SSC. Genes in square brackets are those partially encoded in the IR. Genes in bold emphasize that gene order within the leptosporangiate ferns have been reorganized relative to other plants [37,38].

Most plastome IRs have similar gene content that primarily includes rRNA and tRNA genes [16,22,40]. This is the case even in the ferns that have a reorganized IR [37,38]. Variation from the typical IR gene content has been explained by movement ("ebb and flow") of the IR boundaries into, and out of, the LSC and SSC regions [41]. In our broad sample of land plant plastomes we found several taxa with unique IR boundaries that differ from the basic theme (Fig. 4). In some cases, distantly related taxa have very similar IR boundaries. The liverwort and the two mosses are identical in gene content at both ends of the IR. The two distantly related ferns are also identical to each other. Similarities at only one end of the IR are seen in a wider range of taxa. One end of the LSC is bounded by *trnI-CAU *in all seed-free land plants in our study, except *Selaginella *spp. (because they have lost many or their tRNA genes including *trnI-CAU*) and *Angiopteris evecta*. The SSC is bounded by *ndhF *at one end and *chlL *at the other, in most taxa. This suggests that whereas the ends of the IR clearly ebb and flow in some lineages, in other lineages they appear to be rather stable.

### Phylogenetic Analyses

**Figure 5:**
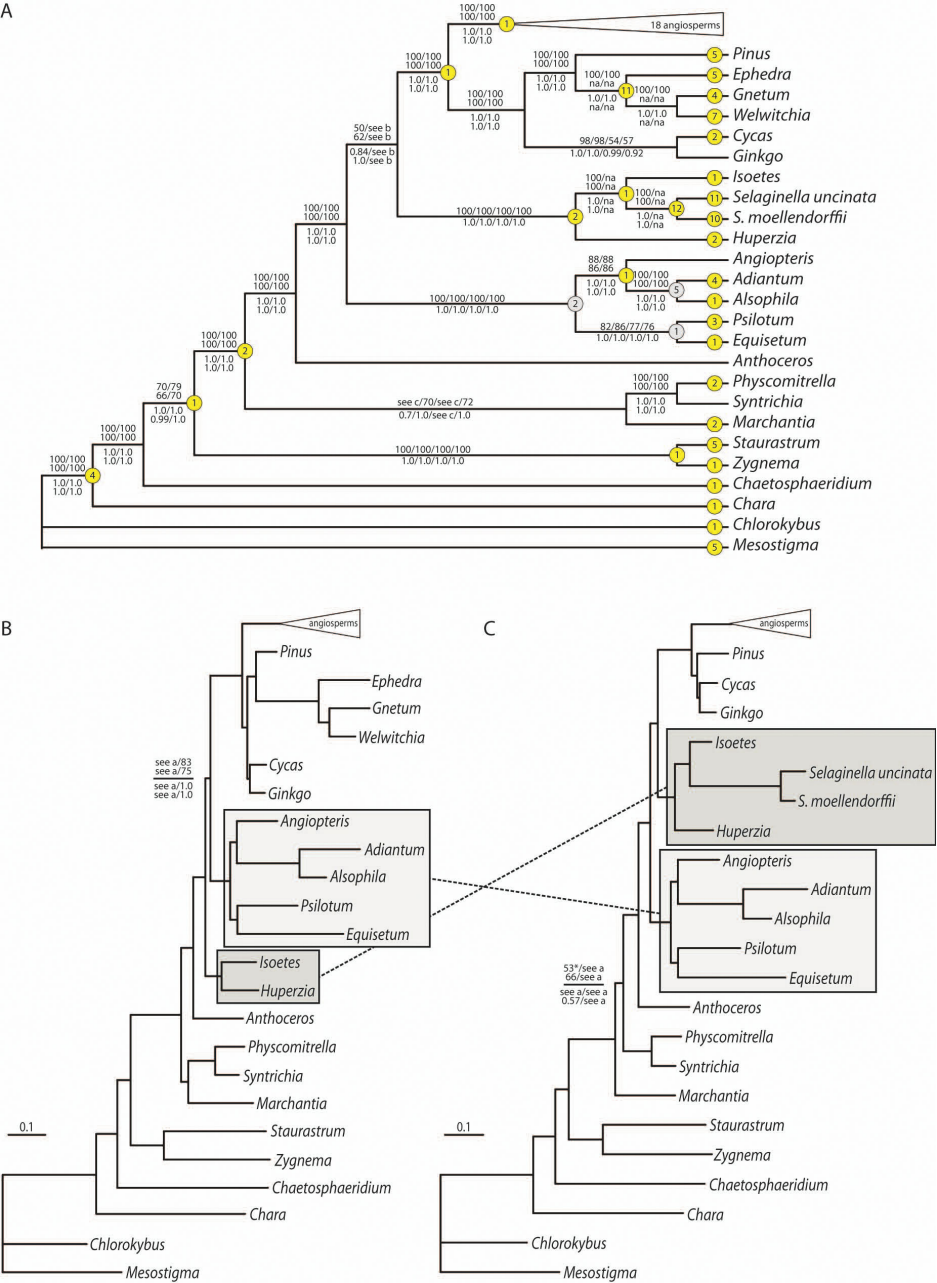
**Phylogenetic results using nucleotide data**. A) Cladogram based on ML analysis (-ln = 473022.24372) of 49 gene sequences from all 43 plastomes sampled (Table 1). Mapped non-homoplastic indels are shown in yellow circles. Numbers in grey circles are indels identified with a more restrictive taxon sampling that were difficult to score across all charophycean green algae and land plants. B) Phylogram based on ML analysis (-ln = 442145.83514) of gene sequences, excluding the two *Selaginella *species. C) Phylogram based on ML analysis (-ln = 436696.62372) of gene sequences, excluding the three gnetophyte taxa (*Ephedra equisetina*, *Gnetum parvifolium *and *Welwitschia mirabilis*). ML phylogenetic results (-ln = 405904.24872) that excluded *Selaginella *spp. and gnetophytes were similar in topology to b (not shown). Numbers above the branches are ML bootstrap proportions and numbers below are Bayesian posterior probabilities in this order: all taxa included/*Selaginella *spp. excluded/gnetophytes excluded/*Selaginella *spp. and gnetophytes excluded. The asterisk (*) in c indicates the ML bootstrap proportion for *Marchantia polymorpha *sister to the remaining land plants found in bootstrap analyses that included all taxa, though the best ML tree recovered a sister relationship of *M. polymorpha *with the mosses *Physcomitrella patens *and *Syntrichia ruralis*. Boxes and dashed lines indicate the relative position of monilophytes (light grey) and lycophytes (dark grey).

We analysed four datasets that included 49 protein-coding genes and each was subjected to Maximum Likelihood (ML) and Bayesian Inference (BI). The datasets differed by the number of taxa included: one data set included all taxa listed in Table 1, another excluded both *Selaginella *species, another excluded all three gnetophyte taxa (*Ephedra equisetina*, *Gnetum parvifolium *and *Welwitschia mirabilis*) and the final excluded both *Selaginella *spp. and all gnetophyte taxa. *Selaginella *spp. and the gnetophytes were selectively included or excluded because of their unusual evolutionary patterns (e.g., plastomes with relatively high G/C content in *Selaginella *spp. and accelerated divergence rates in gnetophytes; [[18,19,35], Banks *et al.* personal communications], which can be problematic for phylogenetic reconstruction). Nearly identical topologies were recovered using nucleotide data regardless of analytical method (ML and BI) or taxon set (exclusion of *Selaginella *spp. and/or gnetophytes) with two exceptions discussed below. A summary including alternate topologies and support values is shown in Fig. 5A-C. Phylogenetic results for angiosperms are shown in Additional file 2.

Phylogenetic relationships among the major bryophyte lineages have been controversial with nearly every possible topology being reported, often with strong support. However, recent convergence in results supports hornworts as sister to vascular plants (as seen here) and liverworts sister to all other land plants [[7,42] and references therein]. Monophyly of the bryophytes has also been proposed using plastome gene data. However this relationship was only found when inferred amino acid sequences or protein plus DNA sequences corrected for A-T bias were analyzed [[15,43,44], Additional file 3]. A sister relationship between mosses and liverworts was proposed previously based on morphological and molecular data. Renzaglia and Garbary [45] concluded that characters related to sperm cell development were compelling evidence for the monophyly of mosses plus liverworts, a clade they referred to as Setaphytes. In a recent analysis that included seven genes from all three DNA compartments (plastid, mitochondria and nuclear) for 192 land plant and green algal taxa, the liverworts were found with strong support as sister to all remaining land plants [12].

In the nucleotide analyses presented here, regardless of taxon set or analytical method, monophyly of land plants was confirmed, as was the sister relationship between the hornwort *Anthoceros formosae *and tracheophytes (e.g., lycophytes, monilophytes, gymnosperms and angiosperms; Fig. 5). More problematic is the position of liverworts among bryophytes as our results show two taxon-dependent placements of the liverwort *Marchantia polymorpha*. In ML and BI analyses that included all taxa or excluded *Selaginella *spp., with or without gnetophytes, *M. polymorpha *was sister to the mosses *Physcomitrella patens *and *Syntrichia ruralis *(Fig. 5A, 5B). The alternative topology with liverworts sister to the remaining land plants was supported in the present study only when the gnetophytes were excluded (Fig. 5C). ML bootstrap proportions were generally weak for this part of the tree regardless of taxon exclusion and BI posterior probabilities were low with the analysis that included all taxa and the analysis that only excluded the gnetophytes. Increased taxon sampling is critical in resolving the pattern of these ancient divergences.

The lycophytes (*Huperzia lucidula, Isoetes flaccida, *with or without *Selaginella *spp.) and monilophytes (*Adiantum capillus-veneris*, *Alsophila spinulosa*, *Angiopteris evecta*, *Equisetum arvense *and *Psilotum nudum*) were each strongly supported monophyletic groups (Fig. 5). However, the relationships of these two clades to each other and to seed plants varied across our analyses. The monilophytes were sister to the seed plants only when *Selaginella *spp. were excluded, with or without gnetophytes (Fig. 5B). Alternatively, the lycophytes were supported (weakly in most analyses) as sister to seed plants when *Selaginella *spp. were included (Fig. 5A, 5C). Strong support was recovered for the monilophyte + seed plant relationship by Qiu *et al.*[12] and this relationship is further supported by the large plastid LSC inversion identified in monilophytes and seed plants and not found in lycophytes (Fig. 2 discussed above) [5,39]. Within the lycophyte clade, the homosporous *H. lucidula *was consistently sister to the heterosporous taxa *I. flaccida *and *Selaginella *spp. (or sister to just *I. flaccida *in analyses where *Selaginella *spp. were excluded).

Strongly supported resolution among the major monilophyte lineages has been elusive, and it was not clear if lack of support was a function of insufficient phylogenetic signal, rapid radiation that is indistinguishable from a true polytomy, or ancient extinctions resulting in long-branch taxa that make phylogenetic reconstruction challenging. Our analyses that used 43,491 nucleotide characters (and several subpartitions of these) revealed a moderately supported sister relationship between *Psilotum nudum *and *Equisetum arvense*. Although these groups are usually not found as sister lineages, this topology is reasonable given the lack of sampling of sister taxa to both genera. More typically, *P. nudum *is found in a lineage that is sister to ophioglossoid ferns [12,13,46], a lineage not sampled in this study, and these two lineages are found sister to the remaining monilophytes (marattioid ferns, horsetails and leptosporangiate ferns). However, a sister relationship between Psilotaceae and Equisetaceae was recently reported in a phylogenetic analysis of morphological characters across vascular plants where five unambiguous character state changes for the two groups were identified [47]. These were primarily characters associated with leaves and organization of the shoot system. Schneider *et al.*[47] did not find the same relationship from an analysis of the same taxa using nucleotide sequences, and they cautioned that the morphological evidence for this sister relationship could well be a function of homoplasy and structural simplification reverting to a pleisiomorphic state, thus causing "concern for a possible misleading bias." Failure of so many studies to resolve relationships among the main lineages of monilophytes is consistent with a rapid radiation (i.e., very short internal branches) rather than lack of phylogenetic signal. Additional data from *Tmesipteris *(a close relative of *Psilotum*), ophioglossoid ferns, and additional *Equisetum *species may help to resolve these nodes.

Some of the deep branching events in the land plant tree are still poorly resolved even with full plastome sequence data now available from most of the major clades. It is likely this is because the intervals of shared history among these major clades were relatively short as compared to their subsequent separate histories, giving rise to extreme asymmetry in branch lengths which causes known problems with inferring the correct tree - particularly using data with a small number of character states such as nucleic acid sequence data [48]. Rare genome structural changes such as gene-order rearrangements have the potential for resolving such short internal branches [49]. Indeed a phylogenetic analysis of plastome gene-order data found liverworts sister to all other land plants and monilophytes sister to the seed plants [50]. However, in that study the lycophytes were not resolved as a monophyletic group as they are here. This topological convergence suggests that a future combined analysis with nucleotide, structural and morphological data may be able to resolve some of the short internal branches.

#### Indels

Many genes contained three base-pair indels with apparent phylogenetic signal. We examined all aligned gene sequences for potential indels across the entire taxon sample, scoring characters as 1 for presence or 0 for absence of a sequence stretch. We scored 152 indels (see Additional files 4 and 5); 40 support the phylogenetic tree (synapomorphic), 99 are unique to a terminal taxon (autapomorphic) and 13 contradict the tree (homoplastic). The synapomorphic and autapomorphic indels are presented in Fig. 5A; the 13 homoplastic indels are not mapped. Indels alone, analyzed by MP, did not provide a well-resolved tree (not shown). However, several nodes were supported by uncontradicted indels; land plants (2 indels), lycophytes (2 indels), *Selaginella *spp. + *Isoetes flaccida *(1 indel), *Selaginella *spp. (12 indels), *Angiopteris evecta *+ *Adiantum capillus-veneris *+ *Alsophila spinulosa *(1 indel), seed plants (1 indel), gnetophytes (11 indels) and angiosperms (1 indel). Angiosperm-specific indels are shown in Additional file 2. Interpretation of indels is dependent on taxon sampling. When subsets of the data were examined more closely we could score additional indels that were difficult to interpret at the broader level. Several such indels were identified; focusing on the monilophytes we found two indels (in *atpA *and *rps4*) uniting the monilophytes; the *rps4 *indel has been reported previously [11]. Five indels in *psbT*, *petG*, *atpA*, *rpl16 *and *rpoB *uniting *Adiantum capillus-veneris *+ *Alsophila spinulosa*, and one in *rpl16 *uniting *Equisetum arvense *and *Psilotum nudum *were also identified (Fig. 5A).

#### Nucleotide composition and codon usage

Codon usage bias is often correlated with genomic nucleotide composition bias but may also be correlated with gene expression, selection and drift [51,52]. The overall G/C content of the genes used in our phylogenetic analyses ranged from 34% in *Marchantia polymorpha *to 54% in *Selaginella uncinata*. Both *S. uncinata *and *S. moellendorffii *were remarkable for their high (greater than 50%) G/C content. Among land plants included in this study, only *M. polymorpha *and the mosses contain less than 35% G/C. The nucleotide composition bias found in many plastomes may result in codon usage biases as well.

The effective number of codons (ENc) is often used as a measure of the amount of codon bias within a gene or genome [53] with values ranging from 20 (very biased) to 61 (very unbiased). Most vascular plants in our study had a value between 46 and 50 (Table 1). Less bias was detected in *Adiantum capillus-veneris *(ENc = 55.12), *Alsophila spinulosa *(52.99), *Selaginella uncinata *(58.30) and *S. moellendorffii *(57.73). More bias was found in bryophytes and charophycean algae, notably *Marchantia polymorpha *(35.25), *Physcomitrella patens *(36.12) and *Syntrichia ruralis *(35.46). These differences may have ramifications for the estimation of phylogenetic relationships among these groups.

## Conclusions

This study illustrates the advantage of whole plastome sequences for studies of plastome architecture and land plant evolution. Nucleotide sequence data from targeted regions have contributed greatly to our understanding of plant phylogeny. However short sequence data can have limitations because they can miss important aspects of genome structure, and nucleotide substitution rates are not always applicable to solving specific evolutionary questions. Restriction site mapping also is limiting because it usually requires a series of cross comparisons every time a taxon is added. Complete plastome sequences provide data that can be used for nucleotide-based analyses, but also can add to a growing database of structural genomic data, including inversions, gene content, and indels. New taxa can be added relatively easily without additional experimental comparisons. Also, changes that might first appear to characterize a large clade might turn out to be more restricted once additional taxa are sampled. For example, some of the unique structural attributes first identified in *Selaginella uncinata *plastome [35] appear to be unique (so far), whereas others are shared with a second plastome sequence of *S. moellendorffii *(Banks *et al.* personal communications). Another important aspect of this type of data is that changes that first appear autapomorphic in small datasets might later emerge as key synapomorphies as additional taxa are sampled. This is illustrated by the series of large inversions in the IR first identified in *Adiantum capillus-veneris *[36,37] but later shown to be shared with the distantly related tree fern *Alsophila spinulosa *[38], and by inference shared among more than 90% of leptosporangiate fern species.

Most of the currently available plastome sequences are from seed plants, with very few available from the presumed sister clade, the monilophytes. Here we have added an additional monilophyte plastome sequence as well as a lycophyte plastome sequence that represent two critical lineages that were not previously sampled (i.e., Equisetaceae and Isoetaceae). We examine multiple aspects of these plastomes to reveal patterns of evolution across land plants. We demonstrate that comparative plastome analysis can provide valuable information about evolutionary processes at the nucleotide, gene and genome scale in early land plants. These data are phylogenetically informative at many levels. Gene rearrangement and indels are informative at deep nodes. Plastome sequences also provide valuable nucleotide data that can be used in studies of ancient and more recent divergences.

Now that plastome sequence data are available for most major lineages of early land plants additional sampling can focus on resolution of poorly supported nodes by increased taxon sampling within these lineages. Although we have one or two representatives of each major group, these often represent lineages containing hundreds or thousands of species. Additional sampling within species-rich lineages may resolve some areas of phylogenetic conflict. As DNA sequencing costs continue to decrease it will become possible to improve sampling and gain further insight into early land plant evolution as well as the patterns and processes that shape the evolution of plastomes.

## Methods

### Taxa

We compared and analyzed complete plastid genomes of 43 taxa, including 37 land plants and six charophycean algae (Table 1). Two of the land plant plastomes are new to this study (herbarium vouchers at WTU): *Equisetum arvense *and *Isoetes flaccida*. For *E. arvense *and *I. flaccida, *tissue preparation, plastid isolation, DNA extraction, cloning, sequencing, and assembly followed Wolf et al.[39]. Annotation was carried out with DOGMA [54] and tRNAscan [55].

### Plastome analyses

Gene maps for representative plastomes were drawn with OGDraw and compared for gene content, gene order and IR boundaries [56]. Codon usage and nucleotide frequencies were determined using CodonW [57].

### DNA alignment

Nucleotide sequences for the 49 protein-coding genes found in all study taxa were extracted from DOGMA-annotated plastomes or from those found in GenBank (Table 1). The hornwort *Anthoceros formosae *and the fern *Adiantum capillus-veneris *both have extensive RNA editing [30,36,37]. Therefore, we used the cDNA sequences rather than genomic sequences for phylogenetic analysis. Each gene was aligned with MacClade v4 [58] and the resulting individual gene alignments were assembled into a single data matrix. A Nexus block was written that identified all unalignable DNA regions, overlapping gene regions, and inferred RNA edited stop codons for exclusion in subsequent phylogenetic analyses. Indels were scored in a separate data matrix as binary characters.

The resulting 49-gene alignment including all codon positions (see Additional file 5) was partitioned into four data sets, 1) one that included all 43 taxa and 30,018 characters, 2) one that excluded both *Selaginella *species (41 taxa and 29,961 characters), 3) one that excluded the gnetophytes *Ephedra equisetina*, *Gnetum parvifolium *and *Welwitschia mirabilis *(40 taxa and 29,871 characters), and 4) one that excluded both *Selaginella *species and all gnetophytes (38 taxa and 29,814 characters). Altering the taxon set changes the number of characters because of lineage specific indels. Nucleotide alignments were translated into amino acids using MacClade assuming the universal code [58]. Ambiguous amino acids resulting from polymorphic nucleotides were treated as missing.

### Phylogenetic Analyses

Maximum Likelihood (ML) and Bayesian Inference (BI) analyses were performed on each of the four nucleotide data sets using PAUP* v4.0b10 [59] and MrBayes v3.1.2 [60-62], respectively. MrModeltest v2 [63] was used to determine the best fitting model of DNA substitution. With the best fitting model (GTR+I+Γ with four rate catagories) an iterative procedure described in Swofford et al.[64] was used to converge on the best fitting model parameters. The resulting model parameters were then fixed for ML analyses and a heuristic search was performed with random taxon addition using TBR branch swapping. Two hundred bootstrap replicates [65] were performed for each data set, each replicate with a single random taxon addition and NNI branch swapping. For BI analyses (also using GTR+I+Γ) two runs each with four markov chains running five million generations were performed. The heated markov chain was sampled every 100^th ^generation. The -ln likelihood scores were plotted against generation number and all samples collected prior to the markov chain reaching stationarity were discarded as burn-in. The remaining samples were summarized using the sumt command in MrBayes. The insertion/deletion matrix was analyzed using the maximum parsimony (MP) criterion in PAUP* as unordered characters with 10 random taxon additions and 200 bootstrap replicates. BI analyses of amino acid data sets were executed in a fashion similar to the nucleotide analyses. We used a fixed model, cpREV+I+Γ, estimated specifically for phylogenetic estimation of plastid-encoded proteins [66]. ML bootstrap analyses of amino acid data were performed in RAxML also using cpREV+I+Γ [67].

## Authors' contributions

PGW, BDM, KSR, RGO, DFM and JLB designed the study. KA and KDEE performed the plastid isolations and flow cytometric sorting. JLB and JVK led the plastome sequencing and assembly. SKH, AMD, PGW and KGK contributed to plastome assembly, finishing and annotation. KGK and JDH performed the phylogenetic analyses and KGK, JDH, PGW and AMD performed plastome comparisons. KGK and PGW wrote a first draft, which final version was completed by KGK, PGW, KSR, JDH, RGO, BDM, KDEE and JLB. All authors read and approved the final manuscript.

## Supplementary Material

Additional file 1 **Plastid *tufA *pseudogene in *Isoetes flaccida*.** The *tufA*-like nucleotide sequence identified in the *Isoetes flaccida *plastome was aligned with the plastid encoded *tufA *sequence of *Chara vulgaris *using ClustalW [85]. An asterisk (*) indicates identical nucleotides and a dash (-) indicates insertion/deletion event (indel). A total 41% nucleotide similarity and 38 indels were identified. Click here for file

Additional file 2 **Angiosperm phylogenetic results using nucleotide data**. The identical angiosperm topology was recovered using nucleotide data regardless of taxon set or analytical method. Nodes with bootstrap proportions (BP) = 100 or posterior probabilities (PP) = 1.0 are not shown (most nodes). Support was generally strong within the angiosperms with a few exceptions (shown). *Nymphaea alba *was always sister to remaining angiosperms, not *Amborella trichopoda*. Support for this was variable (BS = 78-90% depending on taxon set) and this has been addressed better elsewhere (Leebens-Mack et al.[80]). Mapped non-homoplastic indels are shown in yellow circles) and homoplastic indels are not shown. Click here for file

Additional file 3 **Phylogenetic results using inferred amino acid data**. A) Cladogram based on ML analysis using RAxML of 49 inferred amino acid sequences from all 43 plastomes sampled (-ln = 181034.78356; Table 1). RAxML and BI analyses including all taxa as well as those excluding both *Selaginella *spp. and gnetophytes (-ln = 149105.09124) also converged on this topology. Numbers above the branches are ML bootstrap proportions and numbers below are Bayesian posterior probabilities in this order: all taxa included/*Selaginella *spp. excluded/gnetophytes excluded/*Selaginella *spp. and gnetophytes excluded. Support within the angiosperms was generally low in RAxML analyses. *Nymphaea alba *was always sister to remaining angiosperms, not *Amborella trichopoda *(not shown). B) and C) Phylograms based on RAxML analysis excluding either *Selaginella *spp. (-ln = 163523.52584) or gnetophytes (-ln = 166579.56793), respectively. In all phylogenetic analyses of inferred amino acid sequences angiosperms, gymnosperms, lycophytes, monilophytes and bryophytes (in the broad sense) were each monophyletic. However, two different best topologies were discovered depending on taxon set and analytical method. In all BI analyses regardless of taxon set as well as RAxML analyses including all taxa and excluding both *Selaginella *spp. and gnetophytes, the lycophytes were sister to seed plants and monilophytes were sister to all other land plants (including bryophytes) (Additional file 3A). This relationship is inconsistent with most published phylogenies including our nucleotide sequence analyses. Branching order within the monilophytes differed from the topology found using nucleotide data in that *Angiopteris evecta*, *Equisetum arvense *and *Psilotum nudum *formed a paraphyletic grade with respect to *Adiantum capillus-veneris *and *Alsophila spinulosa*; *E. arvense *was sister to the leptosporangiate ferns and *Angiopteris evecta *was sister to the clade containing all other monilophytes. The position of *Angiopteris evecta *is inconsistent with previously published phylogenies [e.g., Pryer et al.[46]]. A different topology was found in RAxML analyses that excluded either *Selaginella *spp. or the gnetophytes (but not both). In these analyses, monilophytes were found sister to seed plants and bryophytes were sister to all other land plants (Additional file 3B, 3C). Although *Angiopteris evecta*, *Equisetum arvense *and *Psilotum nudum *still formed a paraphyletic grade, the relative positions of *A. evecta *and *P. nudum *were switched. Placement of *A. evecta*, *E. arvense *and *P. nudum *had high posterior probabilities (1.0 throughout), but bootstrap support for these clades was low. All nodes within the monilophyte clade had less than 65% support in RAxML bootstrap analyses except for the sister relationship between *Adiantum capillus-veneris *and *Alsophila spinulosa*. Click here for file

Additional file 4 **Insertion/deletion (indel) matrix **Insertion/deletion events (indels) scored across the 49 aligned protein-coding genes used in this study. Characters were scored as 1 for presence or 0 for absence of a sequence stretch. Character state labels (CHARSTATELABELS) indicate in which gene the indel was identified and the position within that gene using the nucleotide alignment in Additional file 5. Click here for file

Additional file 5 **Nucleotide alignment **Alignment of 49 genes from 43 plastomes used for phylogenetic analysis described in the text. Data are interleaved by gene and exclusion blocks are provided at the end of the file. Click here for file
