# Supplementary material for: Complete plastome sequences of Equisetum arvense and Isoetes flaccida: implications for phylogeny and plastid genome evolution of early land plant lineages
Source: BMC Evol Biol. 2010 Oct 23;10:321. doi: 10.1186/1471-2148-10-321 (PMC3087542; doi:10.1186/1471-2148-10-321)
Supplement: Additional file 1 — Plastid tufA pseudogene in Isoetes flaccida. The tufA-like nucleotide sequence identified in the Isoetes flaccida plastome was aligned with the plastid encoded tufA sequence of Chara vulgaris using ClustalW [85]. An asterisk (*) indicates identical nucleotides and a dash (-) indicates insertion/deletion event (indel). A total 41% nucleotide similarity and 38 indels were identified. [file 1471-2148-10-321-S1.PDF]

Additional file 1 - Plastid *tufA* pseudogene in *Isoetes flaccida.*

The *tufA*-like nucleotide sequence identified in the *Isoetes flaccida* plastome was aligned with the plastid encoded *tufA* sequence of *Chara vulgaris* using ClustalW [68]. An asterisk (*) indicates identical nucleotides and a dash (-) indicates insertion/deletion event (indel). A total 41% nucleotide similarity and 38 indels were identified.

```
Chara     ATGGCACAAGAAGTCTTTCAACGTACTAAACCTCATGTAAATATTGGTACTATAGGACAT 60
Isoetes   GTAATTACCTAAATCTCTTTATGACCAAAAAAATAAAGATTAATTCGAAGTACAAAATAT 60
           *        ** *** *  * *  * ***    *   *   *** * * ** *  * **

Chara     GTAGATCATGGAAAAACTACATTAACAGCTGCAATTACAATGACATTAGCAGTTAACAGT 120
Isoetes   CCAATTGAAACGAGATTCTTTCTTATATTCGTCATTACCCTCGCATAATAAGAATATGAA 120
            *  * *    * *       * * *   *  *****  *  *** *  **   *

Chara     ACATGTACTCCTAAGAAATATG-ATGAAATTGATGCTGCTCCAGAAGAAAGAGCACGAGG 179
Isoetes   GAATATATGATTGGGAATGAGGGATATAAAAAATACTTTTTCATAAGTAAAAA-AGGAAG 179
            ** **    *  ***  * * **  **   ** **  * ** *** ** *  * ** *

Chara     TATTACAATTAAT--ACAGCTCATGTGGAATATGAAACAGCTTCACGTCATTATGCT-CA 236
Isoetes   AACTATATTCAAATAATCGTTATTCTAAATCACTTTTCATATTCGTATCGATTTCCAGTC 239
           * ** * * **   *  * *  * *  *  *     **  ***   **  * * *

Chara     TGTAGATTGTCCTGGACATGCAGATTATATAAAAAATATGATAACAGGCGCTGCTCAAAT 296
Isoetes   CGTGAATTGATAAAACGACTCGGAAATCGCGCAAGTTCCTACTGTATGATCTCCACAA-C 298
           **  ****        *  * **        **  *   *    * *  ** * ***

Chara     GGATGGTG---CGATTTTAGTAGTATCTGCTG---CTGATGGACCTATGCCCCAAACTAA 350
Isoetes   GAATAGCGAAACGCTTTTATTTTCGTTTTAAAGGTTCAATTAAAGTGAATTCTCATCCGA 358
          * ** * *   ** ***** *    * *          **  *  *     *  * *  *

Chara     AGAGCACATTTTATTAGCTAAACAAGT------AGGTGTACCTAGTATTG--------TA 396
Isoetes   TCACCTAGCATTACTATTTGTATTTCTTTCGCCAGAATTACATAGTATTGAAATGATTCA 418
            * *     *** **  *  *    *      **   *** ********         *

Chara     GTTTTTTTAA----ATAAAGAAGACCAAGTCGATGATGAAGAGATACTTCAATTGGTAGA 452
Isoetes   ATCTTATCAATTGCATCAGTAATACGAACATAAATTTTGGAGGATGTTCTGGGGGGAGGA 478
           * ** * **    ** *  ** ** **    *   *     ***  *      **  **

Chara     CTTAGAAGTTCGTGAGTCTCTGATCA---ATTACGAATTTC--CTGGAGACAAAGTACCA 507
Isoetes   AATTTTTGTTTGTTCCTTCCAAGGAAGGGAATATAGACTTGTGCTTTGAATCGAGTATGG 538
            *    *** **   *  *     *   * **   * **   **    *   ****

Chara     GTTGTTGCAGGATCTGCTTTAATG--GCTTTACAGGCTTTAACCGAAAAACCGAATACTT 565
Isoetes   GATTTCGCAGTCCCAACCGCCATTCCACATTGTACATTTTCCCCTTGTTTGTATTCGACC 598
          * * * ****   *  *    **    * **  *   ***  **

Chara     TAAGAGGCGAAAATAAGTGGGTAGATAAAATCTATGAGTTAATGGATGCGGTTGATTCTT 625
Isoetes   CTAAAGAGAATAATTCTTACATTCATTATCTCCAACAATAAAGACTTTTTGGAACTTTTT 658
            * **   * ***   *   *  ** *  ** *  * * **    *   *    ** **

Chara     ATATTCCTACCCCTAAAAGAGATA-----TTGAAAAGCCTTTTC---------------- 664
Isoetes   TCCTTTAAACCCATGATGATACTACTCTATTACGAATTCCTTTCGATCCAACTATTTTTA 718
             **   **** * *      **     **   **  * ****

Chara     TAATGCCAATTGAAGACGTATTTTCCATTCA-AGGCCGT-GGTACAGTTGCCACAGGTCG 722
Isoetes   CAGTTTCTCCAAAATAATTAATTCCTATCCCTATGCTATCGATATTGCT-TCACAAACTA 777
           * *  *     ** *  ** ** * ** *  * **  * * **  * *  ****

Chara     AATAGAACGTGGTATCTTAAA-ATTAGGAGATATCGTTGAATTAATTG---GCTTAAACG 778
Isoetes   TGAGGAAACAAAAATCTTATACAGTCGTAGGAAAAGTTTTATCCATCTCATGTTGGGATG 837
              ***      ****** * * * * **  *  ***  **  **     * *   * *

Chara     AAAAAATTCGTAGTACTGTTGTTACAGGACTGGAAATGTTTAGAAG-ACTTTTAGAGCAA 837
Isoetes   CAGGAATTCGATATCCACCTAAT-CATAAATATCTCCGTTTGTATTTACCTCTAGACGAA 896
           *  ******   * *   *  * **  * *      ****  *   ** * ****  **

Chara     GGTTTTGCTGGAGAAAAT-ATTGGTGTACTTTTAAGAGGTATTGAGAAAAAAGATATAGA 896
Isoetes   TACCCAGTAAGGGTAAGCCATTAATGTGAGGCTCGGGATCGCGGGAATGTTACTTTCTGT 956
                *   * * **   ***  ***     *  *       *  *    *  *   *

Chara     AAGAGGAATGGTAATAGCTCAA----CCAGGGAC----AATTGAACCA--CATACCCGTT 946
Isoetes   AAACTAACTCATAATGACTCAAAGTTTCACAGATCTTTAATCTAACTAATTTTAATAATC 1016
          **    * *  ****  *****     **  **     ***  *** *    **    *

Chara     TTGAAGCACAAGTTTATATACTTCGCAAAGAAGAA----------GGAGGTCGTC----- 991
Isoetes   TCTATTCTTTACTCGGTCTTCCTTATTTGAAGAAACTATTGCATTGGGAATCGGCCGAAG 1076
          *  *  *   * *   * * * *       *  **          **   *** *

Chara     -ATTCTCCTTTTTTTGCAGGGTA-----TCGTCCTCAATTC---TTTGTTCGAACTGCAG 1042
Isoetes   AATATTTTTTTGTTCGTAGGATAAATATTCGCTCTCTATTCACCTGTGCCACATCGGTAA 1136
           **  *  *** ** * *** **     ***  *** ****   * **    * * * *

Chara     --ATGTAACTGGTGTAATTGAAGCT-TTTGAATATGACAATGGTGA-TAAAACAAGAATG 1098
Isoetes   TCATATTCTCTGTGTAATCCGTGCGACCCAAATCCATGTGGAGTGAACATAGCGCGCACA 1196
            ** *     *******    **      ***         ****  * * *  * *

Chara     GTTATGCCAGGAGAT---CGAGTTAAAATGATTGTGAATTTAATTTGTCCTATAGCTATC 1155
Isoetes   GGAGTCCCATATTCTACACGGGCTATAAC-ACTCCCACTCT---TTCTCCTGGAGCTGCA 1252
          *   * ***     *   ** * ** **  * *   * * *   ** ****  ****

Chara     GAAA----AGAAAATGAGATTTGC---TATTCGTGAAGGTGGACGTACTATAGGT--GCT 1206
Isoetes   TCAATCTCAAAAGGCTGGCTTTACATTTGTTCGTAACCGCTAAAACCATAGTAATCCGTT 1312
            **    * **     * *** *   * ***** *  *   *     **    *  * *

Chara     GGAGTTGTTTTACAAATATTAGATTCGAC-TCAACTCAAATCAAAAAATAAATAA 1260
Isoetes   GTAGTGGTTTTTCCCTCTGTGACCCATACGTCCAATGGTTCCAGTATTTAAACAT 1367
          * *** ***** *      *       ** ** * *     **  *  **** *
```
