# Supplementary material for: Complete plastome sequences of Equisetum arvense and Isoetes flaccida: implications for phylogeny and plastid genome evolution of early land plant lineages
Source: BMC Evol Biol. 2010 Oct 23;10:321. doi: 10.1186/1471-2148-10-321 (PMC3087542; doi:10.1186/1471-2148-10-321)

Additional file 2 – Angiosperm phylogenetic results using nucleotide data.

The identical angiosperm topology was recovered using nucleotide data regardless of taxon set or analytical method. Nodes with bootstrap proportions (BP)=100 or posterior probabilities (PP)=1.0 are not shown (most nodes). Support was generally strong within the angiosperms with a few exceptions (shown). *Nymphaea alba* was always sister to remaining angiosperms, not *Amborella trichopoda*. Support for this was variable (BS = 78-90% depending on taxon set) and this has been addressed better elsewhere [Leebens-Mack et al. [81]]. Mapped non-homoplastic indels are shown in yellow circles) and homoplastic indels are not shown.

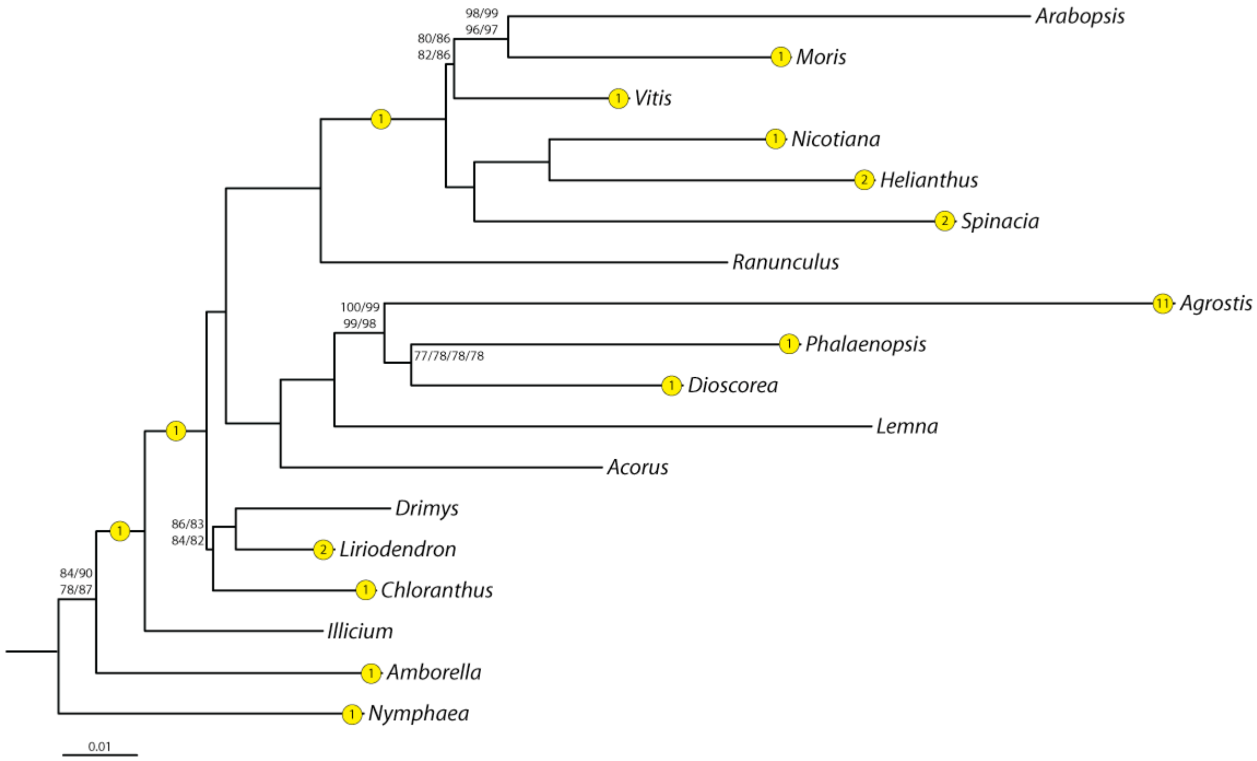

Supplement: Additional file 2 — Angiosperm phylogenetic results using nucleotide data. The identical angiosperm topology was recovered using nucleotide data regardless of taxon set or analytical method. Nodes with bootstrap proportions (BP) = 100 or posterior probabilities (PP) = 1.0 are not shown (most nodes). Support was generally strong within the angiosperms with a few exceptions (shown). Nymphaea alba was always sister to remaining angiosperms, not Amborella trichopoda. Support for this was variable (BS = 78-90% depending on taxon set) and this has been addressed better elsewhere (Leebens-Mack et al.[80]). Mapped non-homoplastic indels are shown in yellow circles) and homoplastic indels are not shown. [file 1471-2148-10-321-S2.PDF]
