# Supplementary material for: Complete plastome sequences of Equisetum arvense and Isoetes flaccida: implications for phylogeny and plastid genome evolution of early land plant lineages
Source: BMC Evol Biol. 2010 Oct 23;10:321. doi: 10.1186/1471-2148-10-321 (PMC3087542; doi:10.1186/1471-2148-10-321)
Supplement: Additional file 3 — Phylogenetic results using inferred amino acid data. A) Cladogram based on ML analysis using RAxML of 49 inferred amino acid sequences from all 43 plastomes sampled (-ln = 181034.78356; Table 1). RAxML and BI analyses including all taxa as well as those excluding both Selaginella spp. and gnetophytes (-ln = 149105.09124) also converged on this topology. Numbers above the branches are ML bootstrap proportions and numbers below are Bayesian posterior probabilities in this order: all taxa included/Selaginella spp. excluded/gnetophytes excluded/Selaginella spp. and gnetophytes excluded. Support within the angiosperms was generally low in RAxML analyses. Nymphaea alba was always sister to remaining angiosperms, not Amborella trichopoda (not shown). B) and C) Phylograms based on RAxML analysis excluding either Selaginella spp. (-ln = 163523.52584) or gnetophytes (-ln = 166579.56793), respectively. In all phylogenetic analyses of inferred amino acid sequences angiosperms, gymnosperms, lycophytes, monilophytes and bryophytes (in the broad sense) were each monophyletic. However, two different best topologies were discovered depending on taxon set and analytical method. In all BI analyses regardless of taxon set as well as RAxML analyses including all taxa and excluding both Selaginella spp. and gnetophytes, the lycophytes were sister to seed plants and monilophytes were sister to all other land plants (including bryophytes) (Additional file 3A). This relationship is inconsistent with most published phylogenies including our nucleotide sequence analyses. Branching order within the monilophytes differed from the topology found using nucleotide data in that Angiopteris evecta, Equisetum arvense and Psilotum nudum formed a paraphyletic grade with respect to Adiantum capillus-veneris and Alsophila spinulosa; E. arvense was sister to the leptosporangiate ferns and Angiopteris evecta was sister to the clade containing all other monilophytes. The position of Angiopteris evecta is in [file 1471-2148-10-321-S3.PDF]

Additional file 3 – Phylogenetic results using inferred amino acid data.

A) Cladogram based on ML analysis using RAxML of 49 inferred amino acid sequences from all 43 plastomes sampled (-ln = 181034.78356; Table 1). RAxML and BI analyses including all taxa as well as those excluding both *Selaginella* spp. and gnetophytes (-ln = 149105.09124) also converged on this topology. Numbers above the branches are ML bootstrap proportions and numbers below are Bayesian posterior probabilities in this order: all taxa included/*Selaginella* spp. excluded/gnetophytes excluded/*Selaginella* spp. and gnetophytes excluded. Support within the angiosperms was generally low in RAxML analyses. *Nymphaea alba* was always sister to remaining angiosperms, not *Amborella trichopoda* (not shown). B) and C) Phylograms based on RAxML analysis excluding either *Selaginella* spp. (-ln = 163523.52584) or gnetophytes (-ln = 166579.56793), respectively.

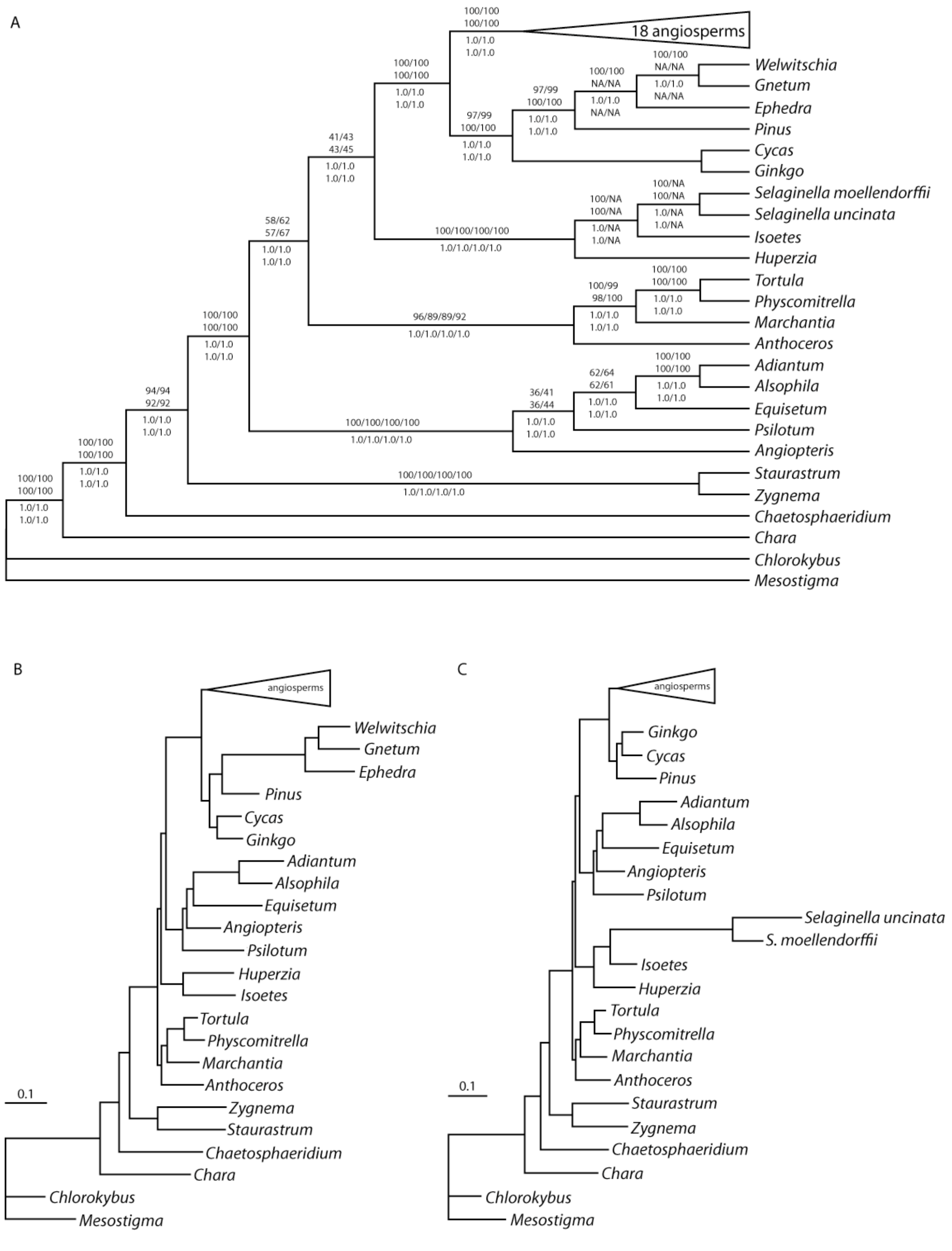

In all phylogenetic analyses of inferred amino acid sequences angiosperms, gymnosperms, lycophytes, monilophytes and bryophytes (in the broad sense) were each monophyletic. However, two different best topologies were discovered depending on taxon set and analytical method. In all BI analyses regardless of taxon

set as well as RAxML analyses including all taxa and excluding both *Selaginella* spp. and gnetophytes, the lycophytes were sister to seed plants and monilophytes were sister to all other land plants (including bryophytes) (Additional file 3A). This relationship is inconsistent with most published phylogenies including our nucleotide sequence analyses. Branching order within the monilophytes differed from the topology found using nucleotide data in that *Angiopteris evecta*, *Equisetum arvense* and *Psilotum nudum* formed a paraphyletic grade with respect to *Adiantum capillus-veneris* and *Alsophila spinulosa*; *E. arvense* was sister to the leptosporangiate ferns and *Angiopteris evecta* was sister to the clade containing all other monilophytes. The position of *Angiopteris evecta* is inconsistent with previously published phylogenies [e.g., Pryer et al. [46]].

A different topology was found in RAxML analyses that excluded either *Selaginella* spp. or the gnetophytes (but not both). In these analyses, monilophytes were found sister to seed plants and bryophytes were sister to all other land plants (Additional file 3B, 3C). Although *Angiopteris evecta*, *Equisetum arvense* and *Psilotum nudum* still formed a paraphyletic grade, the relative positions of *A. evecta* and *P. nudum* were switched. Placement of *A. evecta*, *E. arvense* and *P. nudum* had high posterior probabilities (1.0 throughout), but bootstrap support for these clades was low. All nodes within the monilophyte clade had less than 65% support in RAxML bootstrap analyses except for the sister relationship between *Adiantum capillus-veneris* and *Alsophila spinulosa*.
