# Supplementary material for: Complete plastome sequences of Equisetum arvense and Isoetes flaccida: implications for phylogeny and plastid genome evolution of early land plant lineages
Source: BMC Evol Biol. 2010 Oct 23;10:321. doi: 10.1186/1471-2148-10-321 (PMC3087542; doi:10.1186/1471-2148-10-321)
Supplement: Additional file 4 — Insertion/deletion (indel) matrix Insertion/deletion events (indels) scored across the 49 aligned protein-coding genes used in this study. Characters were scored as 1 for presence or 0 for absence of a sequence stretch. Character state labels (CHARSTATELABELS) indicate in which gene the indel was identified and the position within that gene using the nucleotide alignment in Additional file 5. [file 1471-2148-10-321-S4.PDF]

Additional file 4 – Insertion/deletion (indel) matrix

Insertion/deletion events (indels) scored across the 49 aligned protein-coding genes used in this study. Characters were scored as 1 for presence or 0 for absence of a sequence stretch. Character state labels (CHARSTATELABELS) indicate which gene the indel was identified and the position within that gene using the DNA alignment in Additional file 5.

```
#NEXUS
BEGIN DATA;
    DIMENSIONS  NTAX=43 NCHAR=152;
    FORMAT MISSING=? GAP=- ;
    CHARSTATELABELS

  1  'psaA-25',       52  'ycf4-517',     103  'rps19-319',
  2  'psaA-46',       53  'ycf4-523',     104  'rps19-334',
  3  'psaA-133',      54  'ycf4-538',     105  'rps19-355',
  4  'psaA-586',      55  'ycf4-544',     106  'rps19-424',
  5  'psaA-1546',     56  'rps3-58',      107  'rpl2-127',
  6  'psaA-1555',     57  'rps3-79',      108  'rpl2-232',
  7  'pasA-1891',     58  'rps3-82',      109  'rpl2-838',
  8  'psbA-1030',     59  'rps3-313',     110  'rpl14-79',
  9  'psbA-1036',     60  'rps3-469',     111  'rpl14-277',
 10  'psbA-1057',     61  'rps3-484',     112  'rpl14-331',
 11  'psbE-244',      62  'rps3-487',     113  'rpl16-70',
 12  'psbF-5',        63  'rps3-490',     114  'rpl20-121',
 13  'psbF-22'  ,     64  'rps3-493',     115  'rpl20-139',
 14  'psbH-235',      65  'rps3-637',     116  'rpl20-148',
 15  'psbJ-10',       66  'rps3-646',     117  'rpl20-172',
 16  'psbJ-22',       67  'rps4-49',      118  'rpl20-217',
 17  'psbL-31',       68  'rps4-565',     119  'rpl20-229',
 18  'psbM-4',        69  'rps4-577',     120  'rpl20-322',
 19  'psbN-4',        70  'rps4-580',     121  'rpl36-82',
 20  'psbN-97',       71  'rps4-787',     122  'rpl36-91',
 21  'psbZ-184',      72  'rps4-790',     123  'rpoB-196',
 22  'psbZ-187',      73  'rps7-=244',    124  'rpoB-262',
 23  'rbcL-1405',     74  'rps7-346',     125  'rpoB-269',
 24  'petA-421',      75  'rps7-475',     126  'rpoB-310',
 25  'petA-673',      76  'rps8-4',       127  'rpoB-328',
 26  'petA-682',      77  'rps8-55',      128  'rpoB-349',
 27  'petA-688',      78  'rps8-73',      129  'rpoB-460',
 28  'petA-715',      79  'rps8-97',      130  'rpoB-523',
 29  'petA-730',      80  'rps8-148',     131  'rpoB-1006',
 30  'petA-979',      81  'rps8-202',     132  'rpoB-1033',
 31  'petA-982',      82  'rps8-214',     133  'rpoB-1492',
 32  'petG-103',      83  'rps8-226',     134  'rpoB-1498',
 33  'petG-112',      84  'rps8-244',     135  'rpoB-1621',
 34  'petL-16',       85  'rps8-253',     136  'rpoB-1981',
 35  'atpA-1492',     86  'rps8-262',     137  'rpoB-2137',
 36  'atpB-169',      87  'rps8-265',     138  'rpoB-2137',
 37  'atpB-172',      88  'rps11-112',    139  'rpoB-2347',
 38  'atpB-1452',     89  'rps11-292',    140  'rpoB-2356',
 39  'atpE-4',        90  'rps11-295',    141  'rpoB-2359',
 40  'atpE-7',        91  'rps11-301',    142  'rpoB-2491',
 41  'atpE-58',       92  'rps14-82',     143  'rpoB-2551',
 42  'atpE-166',      93  'rps14-94',     144  'rpoB-2587',
 43  'atpE-344',      94  'rps14-139'     145  'rpoB-2995',,
 44  'atpI-67',       95  'rps14-166'     146  'rpoB-3550',,
 45  'atpI-190',      96  'rps14-295'     147  'rpoC1-1300',,
 46  'atpI-283',      97  'rps14-304'     148  'rpoC1-1489',,
 47  'atpI-289',      98  'rps14-319'     149  'rpoC1-1501',,
 48  'ycf4-433',      99  'rps19-9',      150  'rpoC1-1510',
 49  'ycf4-448',     100  'rps19-13',     151  'rpoC1-1525',
 50  'ycf4-463',     101  'rps19-16',     152  'ycf3-10'
 51  'ycf4-493',     102  'rps19-133',
;
```

```
MATRIX
[                          10        20        30        40        50        60        70    ]
[                          .         .         .         .         .         .         .     ]
Arabidopsis         0001111011101001000010001001101100111101000001010000000001001101000111110011
Morus               0001111011101001000010001001101100111101000001010000000001001101000111110011
Vitis               0001111011101001000010001001101100111101000001010100000001001101000111110011
Nicotiana           0001111011101001000010001001101100111101000001010000000001001101000111110011
Helianthus          0001111011101001000010001001101100111101000001010000000001001101000111110011
Spinacia            0001111011101001000010001001101100111101000001010000000001001101000111110011
Ranunculus          0001111011101001000010001001101100111101000001010000000001001101000111110011
Agrostis            0001111011101001000010001001101100111101010001010000010001001101000111111011
Lemna               0001111011101001000010001001101100111101000001010000000001001101000111110011
Phalaenopsis        0001111011101001000010001001101100111101000001010000000001001101000111110011
Dioscorea           0001111011101001000010001001101100100101000001010000000001001101000111110011
Acorus              0001111011101001000010001001101100111101000001010000000001001101000111110011
Drimys              0001111011101001000010001001101100111101000001010000000001001101000111110011
Liriodendron        0001111011101001000010101001101100111101000001010000000101001101000111110011
Illicium            0001111011101001000010001001101100111101000001010000000001001101000111110011
Chloranthus         0001111011101001000010001001101100111101000001010000000001001101000111110011
Nymphaea            0001111011101001000010001011101100111101000001110000000001001101000111110011
Amborella           0001111011101001000010001001101100111101000001110000000001001101000111110011
Pinus               0101111011101101000010001001101101011101000001110000000001001101000111110011
Ephedra             0010111011101101000000001001101100101001000001111000000001001101000111110000
Gnetum              0011111011101101000010001001101100101001000001111000000001001101000111110010
Welwitschia         0011111011101100000010001001101100101001000001111000000001001001000111000010
Cycas               0001111011101101000010001001101100111101000001110000000001001101000111110111
Ginkgo              000111101110110100001?001001101 1?0111101000001110000000001001101000111?0011
Angiopteris         0001111011101101000010001101101100111101000001110000000001001101000111110011
Adiantum            0001111011101101001010001101101110111101000001110000000001001101000101110011
Alsophila           0001111011101101000011001101101110111101000001110000000001001101000111110011
Psilotum            0001111011101101000010011001101100111100000001110000000011001101100111110011
Equisetum           0001111011001101000010001001101100111101000001110000001001001101000111110011
Isoetes             0001111011101101000010001001101100111101000001110000000001010101000111110011
Selaginella.uncin   0001101111101101010110001001111000111101000010100011000001011101000111110011
Selaginella.moell   0001101011101101110010001001110000111101100100110001100001011101000010110011
Huperzia            0001111011101101000010001001101110111101000001110000000001011101000111110011
Physcomitrella      0001111011101101000010001000101100111111000001110000000001001101000111110011
Tortula             0001111011101101000010001000101100111101000001110000000001001101000111110011
Anthoceros          0001111011101101000010001001101100111101000001110000000001001101000111110011
Marchantia          0001111011101101000010001001101100111100001001110000000000001101000111110011
Chara               0001010011101111000010001000101100111101000001110000000001001101000111110011
Chaetosphaeridium   0001010011101111000010001000101100111101000001110000000001101101010111110011
Staurastrum         0001110001101111000010001000101100111101000001110000000001001111011111110011
Zygnema             0001110010101111000010001000101100111101000001110000000001001101010111110011
Chlorokybus         1001010011010111000010001000101100111101010001110000000001001100000111110011
Mesostigma          1001010011010111000010000000001100111101010001110000000001001100000111110011

[                          80        90        100       110       120       130       140       150]
[                          .         .         .         .         .         .         .         .  ]
Arabidopsis         010000010110110100011111000000111001001110110011000110001001011011111111000101
Morus               010100110110110100011111000000111001001110110011000110001001011011111111000101
Vitis               010100010110110100011111000000111001001110110011000110001001011011111111000101
Nicotiana           010100010110110100011111000000111011001110110011000110001001011011111111000101
Helianthus          010100010110110100011111000000111001001110110011001110001001001011111111000101
Spinacia            010100010110110100011111000000111000001110110011000110001001011011111111000100
Ranunculus          010100010100110100011111000000111001001110110011000110001001011011111111000101
Agrostis            010101011100 0??1100111100010001101010011101100110001100111010111111111000101
Lemna               010100010100110100011111000000110001001110110011000110001001011011111111000101
Phalaenopsis        010100010000110100011111000000110001001110110011000110001001011011111111000101
Dioscorea           010100010100110100011111000000110001001110110011000110001001011011111111000101
Acorus              010100010100110100011111000000110001001110110011000110001001011011111111000101
Drimys              010100010100110100011111000000110001001110110011000110001001011011111111000101
Liriodendron        010100010100110100011111000000110001001110110011000110001001011011111111000101
Illicium            010100010100110100011111000000110001001110010011000110001001011011111111000101
Chloranthus         011100010100110100011111000000110001001110110011000110001001011011111111000101
Nymphaea            010100010100110100011111000000110001001110010011000110001001011011111111000101
Amborella           010100010100110100011111000000110001001110010011000110001011011011111111000101
Pinus               010100010100110000011111000000110001001110010011000110001001011011111111100101
Ephedra             0101000001001 0?1000111111001001110010011100100010100100010010100010011010001
Gnetum              0101000001001 0?1000111011001001100010011100100010100100000010100010001000111
Welwitschia         0101000001001 0?1000110111101101100010011100100010100100010010100010011000101
Cycas               010100010100110100011111000000110001101110010011000110001001011011111111000101
Ginkgo              01010001010011010001111100000?110001001110010011000110001001011011111111000101
Angiopteris         010100010100110100011111000000010001001110010011000110001001011011111111000101
Adiantum            100100010100110100011111000000010001001110010011000110001001011011111111000101
Alsophila           010100010100110100011111000000010001001110010011000110001001011011111111000101
Psilotum            010000010100110100011111000000010001001110010011000110001001011010111111000101
Equisetum           010100010100110100011111000000010001001110010011000110001001011011111111000101
Isoetes             010100010101110100011111000000010001001110010011000110001001011111111110000101
Selaginella.uncin   010100010101110101100111000000010001000001011011000110001000111111111110000101
Selaginella.moell   010100010101110100011111000000010001011111001011000110001000111111111110000101
Huperzia            010100010100111100011111000000010001001110010011100110001001011011111110000101
Physcomitrella      010100010100110100011111000000010001001110010010010110001001011011111111000101
Tortula             010100010100110100011111000000010001001110010011000110001001011011111111000101
Anthoceros          010100010100110100011111000000010001001110010011000110001001011011111111000101
Marchantia          010100010100110100011111000000010001001110010011000110001001011011111111000101
Chara               010110010100110100011111000000010001001110010011000110001001011011111111000101
Chaetosphaeridium   010100010100110100011111000000010001001110010011000110001001011011111111000101
Staurastrum         010100010100110100011111000000010001001110010011000101101001011011111111000101
Zygnema             010100010100110100011111000000010001001110010011000100001001011011111111000101
Chlorokybus         010100010100110100011111000000010001001110010011000110001001011010111111001101
Mesostigma          010100010100110100011111000001000001001110010111000010001001011010111111000101
;
END;
```
